# Multiomics of three hematological malignancies in a patient reveal their origin from clonal hematopoietic stem cells

**DOI:** 10.1038/s41408-023-00892-w

**Published:** 2023-08-09

**Authors:** Sylvain Mayeur, Anne Molitor, Laurent Miguet, Lucie Rigolot, Lydie Naegely, Tristan Stemmelen, Sébastien Meyer, Elise Toussaint, Laurent Vallat, Alice Eischen, Marie-Pierre Chenard, Manuela Tavian, Seiamak Bahram, Raphael Carapito, Alina Nicolae

**Affiliations:** 1 Laboratoire d’ImmunoRhumatologie Moléculaire, Plateforme GENOMAX, INSERM UMR_S 1109, Faculté de Médecine, Fédération Hospitalo-Universitaire OMICARE, ITI TRANSPLANTEX NG, Fédération de Médecine Translationnelle de Strasbourg (FMTS), Université de Strasbourg, Strasbourg, France; 2 Département de Pathologie, Hôpital de Hautepierre, Hôpitaux Universitaires de Strasbourg, Strasbourg, France; 3 Laboratoire d’Hématologie, Pôle de Biologie, Hôpital de Hautepierre, Hôpitaux Universitaires de Strasbourg, Strasbourg, France; 4 INSERM, IRFAC / UMR_S 1113, ITI InnoVec, FHU ARRIMAGE, FMTS, Université de Strasbourg, Strasbourg, France; 5 Service d’Hématologie, Hôpitaux Universitaires de Strasbourg, Strasbourg, France; 6 Laboratoire d’Immunologie, Plateau Technique de Biologie, Pôle de Biologie, Nouvel Hôpital Civil, Strasbourg, France

**Keywords:** Oncogenesis, Cancer stem cells

**Dear Editor**,

Multiple hematological malignancies can occur simultaneously or sequentially in a single patient. They are challenging to diagnose and their prevalence is likely underscored in the absence of systematic pathological review of refractory or relapse disease. Several non-mutually exclusive mechanisms have been proposed to explain their oncogenesis: (1) immune dysregulation allowing divergent neoplastic clones’ expansion linked or not to Epstein-Barr virus (EBV) infection; (2) clonal hematopoiesis (CH) with genomic alterations in hematopoietic stem and progenitor cells (HSPCs); (3) chronic antigenic stimulation; (4) exposure to genotoxic stress from cytotoxic drugs and radiation therapy [1–3]. Most reported cases associate two hematological malignancies and co-existence of three malignancies are very rare. To our knowledge, occurrence of B-, T- and myeloid neoplasms in a patient has been reported only twice as metachronous events [4, 5].

Here, we present a 62 years-old Caucasian man smoker who presented with severe dyspnea. Except hypertension, his medical history was unremarkable. Imaging studies revealed pericardial effusion and left ventricle tumoral invasion (Supplementary Fig. 1A) with mild hypermetabolism of left adrenal gland and anterior mediastinal lymph nodes (LNs). Laboratory findings showed anemia (hemoglobin 10.9 g/dl) and hypereosinophilia (1.3 x10^9^/L). LDH and β2-microglobulin levels were normal and the virological workup was negative. Pericardiocentesis yielded 1.7 L of sero-hematic fluid. It was involved by a CD30+ mature T cell lymphoma with cytotoxic phenotype consistent with ALK negative anaplastic large T cell lymphoma (ALK- ALCL) based on cytological assessment, immunophenotyping and PCR analysis of T-cell receptor gamma gene (*TRG*) rearrangements (Supplementary Fig. 1 and Supplementary Table 1). Bone marrow (BM) aspirate and peripheral blood (PB) smears showed a mild excess of monocytes and eosinophils, without other cytological changes or lymphoma involvement. The patient received multiple lines of treatment including 2x Bv-CHP (brentuximab vedotin, cyclophosphamide, doxorubicin, and prednisone), 2x Bv-DHAC (dexamethasone, cytarabine, carboplatin) and 1x GVD (gemcitabine, vinorelbine, doxorubicine) with no favorable response. Upon treatment, he developed aortitis, mesenteric panniculitis, CMV ulcerative recto-sigmoiditis with subsequent septic shock. Enlargement of right axillary LNs was documented at 15 months after the initial presentation. An excisional LN biopsy showed beside ALK- ALCL persistence, a diffuse large B-cell lymphoma (DLBCL-NOS) that was further confirmed by a monoclonal immunoglobulin kappa locus (*IGK*) rearrangement and an acute myeloid leukemia with monoblastic/monocytic differentiation (AML-M5) (Fig. 1 and Supplementary Table 1). PB and BM aspirates showed no lymphomatous involvement, but 90% and 47% blasts, respectively, of mostly monoblasts consistent with AML-M5 (Supplementary Fig. 2). The BM karyotype showed 4 independent clones: 46,XY,add(10)(q21)[8]/45,sl,-21[4]/ 44,X,-Y,der(2;16)(q10-p10)[4]/45,sl,+r[6]/ 44,X,-Y,-21[7]/ 47,XY, + 8[5]/46,XY[14] and absence of *KMT2A* gene rearrangement by FISH analysis. The patient died soon after the diagnosis of concomitant T-, B-cell lymphomas and AML.

**Fig. 1:**
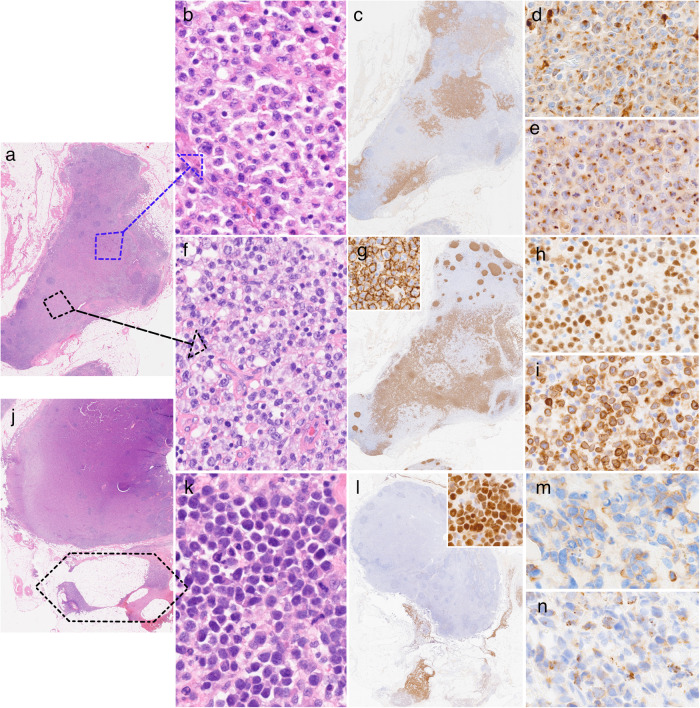
Concomitant ALK- ALCL, DLBCL-NOS and AML-M5 in a single lymph node (LN) resection specimen. **a** Low power examination revealed partial effacement of LN architecture by two neoplastic components (blue arrow - zoom in on LAM-M5 and black arrow - area of DLBCL). **b** Proliferation of medium sized blasts with irregular nuclei, fine chromatin, inconspicuous nucleoli, and moderate amount of pale cytoplasm. They expressed CD56 (**c**), CD68 (**d**) and lysozyme (**e**) consistent with monoblastic/monocytic differentiation (LAM-M5). **f** Other LN areas were rich in atypical lymphocytes often with centroblastic appearance. They were of B-cell lineage positive for CD20 (low and high power - inset) (**g**), PAX5 (**h**) and CD79a (**i**). **j** Another section of the same specimen showed diffuse effacement of LN parenchyma mainly by the blastic infiltrate. A third neoplastic component was seen restricted to the perinodal adipose tissue (black area). **k** There were dense sheets of large, mitotically active lymphocytes, with round, irregular or kidney-shaped nuclei (“hallmark” cells) and moderate amount of eosinophilic cytoplasm. They strongly expressed MUM1 (low and high power - inset) (**l**) and partially CD2 (**m**) and granzyme B (**n**). By PCR, they shared the same TRG rearrangements with pericardial neoplastic T-cell infiltrate (not shown). Stains original magnification x10 (low power images) and x400 (high power images and insets), H&E hematoxylin and eosin.

Given this unusual association, we sought to characterize molecularly the three hematological malignancies. Pericardial and LN ALK- ALCL, DLBCL-NOS, non-leukemic and AML-M5 BM aspirates and sorted CD34+ HSPCs were analyzed by whole exome sequencing (WES) and targeted deep sequencing (TDS). Additionally, normal cartilage served as germline control for WES analysis. A total of 1150 nonsynonymous somatic mutations involving 1047 genes were detected by WES. Single-nucleotide variants represented 95% of mutations and included 87% missense, 5% nonsense, 4% in-frame insertions/deletions and 3% splice-site variants. A myriad of neoplasm-specific mutations was observed, with a total of 543 variants involving 503 genes in pericardial and nodal ALK- ALCL, 355 variants involving 343 genes in DLBCL-NOS and 200 variants involving 186 genes in AML-M5 (Fig. 2a and Supplementary Table 2). Focusing on common mutations, two *TET2* mutations (p.Q1524* and the splice site c.3955-1 G > A) were shared between all three hematological malignancies. Remarkably, they were also encountered in the BM without cytological atypia or clonal T-cells and sorted CD34+ HSPCs, indicating a common clonal HSPC origin of these three neoplasms. Both *TET2* variants had variant allele frequencies (VAF) > 25% across all samples. The three neoplasms also showed TP53 p.V216E mutation at a VAF ranging from 2% to 4% by TDS. Additionally, nodal and pericardial ALK- ALCL shared two deleterious mutations involving *TP53* (p.V173M) and *LYN* proto-oncogene (p.N416T) (Fig. 2b). Other mutations involving *SND1*, *BAX, XPO1* and *KDM6A w*ere observed in the LN ALK- ALCL, but not in pericardium and the reverse was seen for *CCR7, CDKN2C* and *BMPR1A* gene mutations. The DLBCL-NOS component was characterized by genomic alterations in *KRAS* (p.G13C), *STAT6* (p.W515L), *CREBBP*, *PIM1, BCL6, EBF1* and *ATM* genes, known to be dysregulated in this lymphoma [6, 7]. In addition, it shared with AML-M5 an *ANXA11* missense mutation (p.A357T). Other hits specific to AML-M5 sample included mutations in *JAK3* (p.P132Q, VAF: 33%) and *PPM1D* (p.A370S, VAF: 30%), a negative regulator of DNA damage response. It also harbored multiple *KMT2D* and two *NF1* genomic alterations. Furthermore, AML-M5 shared a *PABPC1* (p.G123C) mutation with the pre-leukemic BM aspirate. This poly(A)-binding protein gene is reported as a cancer gene in COSMIC and its alterations were described as novel CH mutations in patients with lung cancer [8]. A subset of cytologically non-atypical BM cells displayed additionally genomic alterations in *HNF1A*, *MST1*, *FAT3* and *RBMX* genes (VAF range 11%-17%). These mutations were observed neither in the CD34+ HSPCs, nor in the three hematological neoplasms. Moreover, TDS identified a *SRSF2* p.P96L missense mutation in CD34+HSPCs and pre-leukemic BM sample at VAF of 1% and 2%, respectively. Lastly, CD34+HSPCs showed mutations in *GCLC*, a key glutathione metabolic enzyme highly expressed in normal HSPCs [9] and *UFL1*, a Ufm1 E3 ligase essential for hematopoietic stem cell survival and function [10]. The presumptive oncogenic steps involved in B-, T- and myeloid neoplasms development are shown in Fig. 2c.

**Fig. 2:**
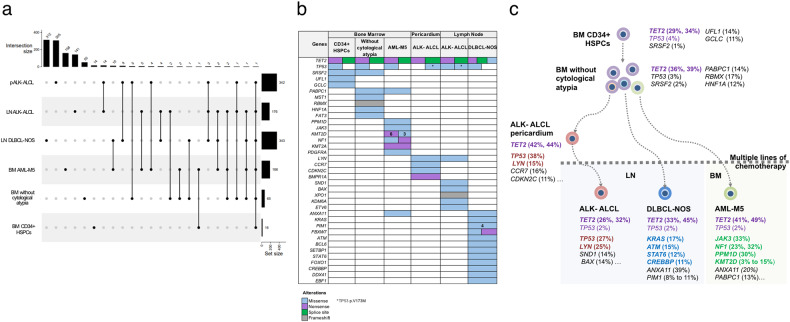
Molecular landscape of the three hematological malignancies and presumptive oncogenic steps involved in their development and concurrence. **a** Upset plot representation of the total number of mutated genes by WES. Horizontal bars represent the total number of genes mutated in each sample. Subset of gene(s) mutated in a unique sample or in multiple samples is represented by a dot and a line, respectively. Vertical bars represent the number of genes mutated in each sample. **b** Oncoplot representation of selected mutated genes in all samples by WES and TDS. The number of multiple somatic variants is indicated. **c** All three neoplasms shared two *TET2* mutations at high allele burden with CD34+ HSPCs and BM aspirate without cytological atypia. Subsequent acquisition of additional hits particular to each neoplasm were likely responsible for divergent malignant progression. LN - lymph node, BM – bone marrow, HSPCs - hematopoietic stem and progenitor cells, ALCL – anaplastic large cell lymphoma, DLBCL-NOS – diffuse large B-cell lymphoma not otherwise specified, AML – acute myeloid leukemia.

In addition to genomics, we performed spatial transcriptomic profiling of each neoplastic component in the LN. Based on morphology and multiplex immunophenotyping, 39 Regions of Interests (ROI) including 7 ALK- ALCL, 11 DLBCL, 12 AML-M5, 5 residual non neoplastic paracortical regions and 4 non-atypical B-cell lymphoid follicles were analyzed (Supplementary Fig. 3a–c). Hierarchical clustering based on normalized sequencing read counts allowed clear separation of the three neoplastic components, confirming their distinctive nature (Supplementary Fig. 3d). The expression data were further validated by the correlation of *TP53*, *IRF4/MUM1*, *MS4A1/CD20*, *PAX5*, *BCL6* and *CD56* mRNA transcripts level and their protein expression by immunohistochemistry (Pearson R = 0.7659, *P* = 0.0002 Supplementary Fig. 4). A total of 18,678 genes were quantified by spatial transcriptomics. Comparatively to the non-neoplastic sample group, 5275, 9484 and 16,464 genes were significantly differentially expressed in the DLBCL, ALK- ALCL and AML-M5 groups, respectively (Supplementary Table 3). Among all differentially expressed genes, we identified 1680, 3073 and 9721 genes set signature specific to the DLBCL, ALK- ALCL and AML-M5 groups, respectively. Using Gene Set Enrichment Analysis, the ALK- ALCL signature was characterized by upregulation of genes related to “G1 to S cell cycle control” and “Retinoblastoma Gene in cancer” pathways (Supplementary Table 4). Downregulated genes in ALK- ALCL signature were predominantly related to “Type II interferon signaling”, “Chemokine signaling” and “Integrin-mediated cell adhesion” pathways. With respects to the AML-M5 signature, upregulated genes were enriched in “Chemokine signaling” and “Integrin-mediated cell adhesion” pathways. Due to a low number of differentially expressed genes, no pathway was significantly enriched in the DLBCL signature. Using the Partial Least Squares Discriminant Analysis regression associated with our adapted “gene shaving” method, Top5 genes for which transcriptomic profiles best differentiate neoplasms from each other were obtained (Supplementary Fig. 5a and Supplementary Table 5). Interestingly, six genes of the Top5 gene lists (*NFKB2*, *JAK3*, *IL21R*, *STAT3*, *LCK* and *RHOA*) are known cancer census genes and are critical players in JAK/STAT and NFkB signaling pathways (Supplementary Fig. 4b and Supplementary Table 6 for the list of Top100 discriminating genes).

One of the main finding of this work is the establishment of clonal relatedness of three hematological malignancies to *TET2* mutated CD34+HSPCs. So far, CH involvement in the pathobiology of cytotoxic T-cell neoplasms has been only suspected, with recurrent *TET2* and *DNMT3A* mutations documented in nodal cytotoxic peripheral T-cell lymphomas (TCL), some with history of B-cell lymphomas or MDS [11]. Our study presents evidence of cytotoxic TCL origin from *TET2* HSPC mutants. The large *TET2* clone(s) size likely conferred the propitious ground [12], however, additional hits were required for these neoplasms to emerge. Both pericardial and nodal ALK- ALCL showed a high allele burden of TP53 p.V173M mutation (VAF: 38% and 27%, respectively), which is recognized as highly oncogenic [13]. In addition, they shared a deleterious LYN p.N417T variant. While not expressed by the normal T-cells [14], Lck/yes-related protein tyrosine kinase (*LYN*) gene mutations or *LYN* mRNA overexpression have been occasionally reported in angioimmunoblastic T-cell lymphoma and ALK + ALCL, respectively [15]. Although *LYN* alterations rarely appear to be a primary initiating event in leukemia/lymphoma, their role in oncogenic signaling cascades in cancer/leukemia is undeniable [16].

To conclude, to our knowledge this is the first report documenting co-occurrence of three lineage distinct hematological neoplasms in a single specimen and demonstrating their clonal relatedness to *TET2* mutated HSPCs. The high-risk CH together with the genotoxic stress induced by chemotherapy most probably contributed to their oncogenesis. Besides *TET2* mutations, B-, T-cell lymphomas and AML showed divergent genomic evolution with acquisition of subsequent neoplasm-specific hits and displayed specific gene signatures by spatial transcriptomics.

### Supplementary information

Supplemental Material

Supplemental Figure 1

Supplemental Figure 2

Supplemental Figure 3

Supplemental Figure 4

Supplemental Figure 5

Supplemental Table 1

Supplemental Table 2

Supplemental Table 3

Supplemental Table 4

Supplemental Table 5

Supplemental Table 6

Supplemental Table 7

## Data Availability

Raw exome sequencing data were deposited as FASTQ files at the National Center for Biotechnology Information’s Sequence Read Archive (accession no. PRJNA935278). Raw RNA-seq data have been deposited in the EMBL-EBI ArrayExpress archive (accession no. E-MTAB-12742).
