# Supplementary material for: Multiomics of three hematological malignancies in a patient reveal their origin from clonal hematopoietic stem cells
Source: Blood Cancer J. 2023 Aug 9;13(1):118. doi: 10.1038/s41408-023-00892-w (PMC10412639; doi:10.1038/s41408-023-00892-w)
Supplement: Supplementary file 1 — Supplemental Material [file 41408_2023_892_MOESM1_ESM.docx]

**Supplemental data**

**Multiomics of three hematological malignancies in a patient reveal their origin from clonal hematopoietic stem cells**

**List of Supplemental Tables provided as excel files**

**Table 1.** Immunophenotype and EBV status of pericardial and lymph node ALK- ALCL, DLBCL-NOS and AML-M5

**Table 2.** All coding somatic variants detected by WES, TDS after filtering and manually validated somatic variants

**Table 3.** Differential expressed genes in each neoplastic component versus non-neoplastic lymphoid tissue using spatial transcriptomics

**Table 4.** Gene set enrichment analysis of ALK-ALCL and AML-M5 components using Wikipathway cancer database

**Table 5.** TOP5 gene list allowing distinction between the three neoplasms

**Table 6**. TOP100 gene allowing distinction between the three neoplasms using partial least squares discriminant analysis (PLS-DA)

**Table 7.** Antibodies used for immunohistochemistry and flow cytometry analysis

**List of Supplemental Figures which legends are provided within this document**

**Figure 1.** Pericardial ALK- ALCL.

**Figure 2.** Cytology and phenotype by flow cytometry of AML-M5 in the bone marrow aspirate

**Figure 3.** Spatial transcriptomics of the three hematological malignancies in the lymph node specimen

**Figure 4.** mRNA and protein expression correlation in ALK-ALCL, DLBCL-NOS and AML-M5

**Figure 5.** Transcriptomic signatures of the three hematological malignancies

**Methods**

**Tumor samples**

The patient’s cytological pericardial sample was received in the frame of Lymphopath French registry. The patient was subsequently admitted to our institution, and he consented for the use of cytological and tissue samples for research purpose, including germline analysis. Samples were collected and processed following standard ethical procedures (Declaration of Helsinki). Sorted CD34+ hematopoietic stem and progenitor cells (HSPCs), uninvolved and leukemic Bone Marrow (BM) aspirates, Peripheral Blood (PB) smears, pericardial fluid, and neoplastic Lymph Node (LN) specimen were carefully reviewed.

**Immunohistochemistry (IHC) and *in situ* hybridization studies**

IHC was performed on formalin-fixed and paraffin-embedded (FFPE) pericardial cell pellet and LN sections following routine protocols on a Ventana BenchMark Ultra automated stainer (Ventana, Tucson, AZ, USA). An extensive antibody panel was used (Supplemental table 7)**.** FFPE samples were evaluated for presence of EBV by *in situ* hybridization using INFORM EBER Probe (Ventana Medical Systems, Roche Diagnostics GmbH, Mannheim Germany) supplied by Ventana on the same stainer.

**Flow cytometry (FC)**

Multiparameter FC analysis was performed on BM aspirate and PB at the time of AML-M5 diagnosis using the panel of antibodies listed in the Supplemental table 7 and according to standard methods on a FACScanto^TM^ instrument (Becton Dickinson, Franklin Lakes, NJ, USA) [1].

**Hematopoietic stem and progenitor cells isolation**

An apheresis blood collection bag sample of 6.8 x 10^8^ cells harvested from the patient after a plerixafor treatment has been used to isolate HSPCs. Mononuclear cells were pre-isolated by density gradient centrifugation (FicollPaque Plus, VWR International, Radnor, PA, USA) before FACS sorting (FACSMelody Cell Sorter, BD Biosciences, Franklin Lakes, NJ, USA). The antibodies used included: PE-conjugated anti-CD34 (clone 581, BD Biosciences, Franklin Lakes, NJ, USA), APC-H7-conjugated anti-CD45 (clone 2D1, BD Biosciences, Franklin Lakes, NJ, USA) and 7AAD (A9400, Sigma Aldrich, Saint Louis, MI, USA). Based on CD34^+^CD45^-^7AAD^-^ phenotype, 2.4 x 10^6^ HSCPs were isolated with a post-isolation purity over 95% and were stored as dry cell pellet at -80°C.

**DNA extraction**

GeneRead DNA FFPE kit (Qiagen, Hilden, Germany) allowed for DNA extraction from FFPE pericardial cell-block, macrodissected B- and T-cell lymphoma components from the LN and macrodissected normal cartilage (for germline DNA) from a non-diagnostic BM biopsy. QIAamp DNA mini-Kit (Qiagen, Hilden, Germany) and Maxwell RSC Buffy Coat DNA kit (Promega, Madison, USA) were used for DNA extraction from fresh leukemic BM aspirate and respectively, uninvolved BM aspirate and CD34+HSPCs according to the manufacturers’ recommendations.

**PCR for T-cell receptor and immunoglobulin gene rearrangements**

Clonality was studied on apheresis blood collection, pericardial effusion cell-block and macro-dissected B- and T-cell lymphoma regions of the LN by multiplex PCR assays targeting T-cell receptor gamma genes (TRG), immunoglobulin heavy chain (IGH) and kappa chain (IGK) genes rearrangements using Biomed-2 primers as previously reported [2].

**Whole exome sequencing (WES)**

WES was performed on seven samples including: pericardial and lymph node ALK- anaplastic large cell lymphoma (ALK-ALCL), diffuse large B-cell lymphoma, not otherwise specified (DLBCL-NOS) from the LN, uninvolved and acute monoblastic leukemia (AML-M5) bone marrow aspirates, CD34+ HSPCs and normal cartilage. Genomic DNA was quantified using a Qubit instrument and the dsDNA BR Assay kit (Thermo Fisher Scientific, Waltham, MA, USA).

Sequencing libraries were prepared with the Twist Human Core Exome Kit (Twist Bioscience, San Francisco, CA, USA) following the manufacturer’s recommendations. Paired end (2 × 75 bp) sequencing was performed on a NextSeq500 sequencer (Illumina, San Diego, CA, USA). Sequences were mapped to the hg19 reference genome using bwa v0.7.17 [3]. Sequence variants were called using Mutect2 v4.1.8.0 [4] and Vardict v1.8.2 [5].

Variants were annotated using VEP (version 94) [6] and filtered as follows: depth of coverage in tumor and corresponding normal samples >10, depth of coverage of alternative bases in tumor ≥5 and in non-tumoral control = 0. Variants reported with a frequency >1% in the 1000Genomes Project or gnomAD databases were discarded. We focused only on protein-altering variants (missense, nonsense, splice site variants, start and stop gain/loss and coding indels). The impact of mutations on protein function was assessed by consulting public databases, such as COSMIC, variant pathogenicity predictors (SIFT, PolyPhen, LRT, MutationTaster and FATHMM) and the literature.

**Targeted deep sequencing (TDS)**

To confirm WES results, 6 samples (except normal cartilage) were analysed by TDS using a customized panel covering mutational hotspots in 103 genes relevant to myeloid and lymphoid malignancies [7-9] used in routine diagnosis. Briefly, libraries were prepared with targeted Agilent XTHS capture panel (Agilent, Santa Clara, CA, USA) following the manufacturer’s recommendations. Paired end sequencing (2 x 150 bp) was done on Illumina NextSeq550 (Illumina, San Diego, CA, USA) using 500/550 Mid Output kit v2.5. Bioinformatics analysis (mapping, calling and annotation) were performed with STARK (version 0.9b [10]; https://github.com/bioinfo-chru-strasbourg/STARK) using hg19 as reference genome. The variant call fille has been manually analysed. Only non-polymorphic non-synonymous coding variants present at an allelic frequency >2% and a sequencing depth >300 reads were reported. Retained variants were all visually inspected using Integrative Genomics Viewer (IGV, Broad Institute) and Alamut Visual^tm^ (V2.15 Feb. 2020).

**Spatial transcriptomics**

To explore the transcriptome profiling of T-, B-cell lymphomas and AML in the FFPE LN specimen, NanoString GeoMx Digital Spatial Profiler technology with the GeoMx Human whole transcriptome atlas (Nanostring, Seattle, WA, USA) was used [11]. Guided by both H&E staining and immunofluorescent markers, a final set of 39 regions of interest (ROIs) including 7 ALK- ALCL, 11 DLBCL-NOS, 12 AML-M5, 5 residuals non-neoplastic paracortical regions and 4 non-atypical B-cell lymphoid follicles were analyzed. Only targets above the limit of quantification in at least 5% of the ROIs were considered and normalization was performed as previously described [12] to obtain a final list of 18,678 genes. For differential expression (DE) analysis, we used Limma-voom from the edgeR package, version 3.38.4 [13].

**Functional transcriptomic enrichment analyses**

From the differential analysis provided by edgeR, fold-changes against controls and associated p-values were obtained. All fold-changes were log-transformed and unsignificant results were set to 0. Signature gene sets were defined as follows : $G_{c}=\{g : \sigma\left( \bar{F_{g,c}} \right)\neq0, \sigma\left( \bar{F_{g,d \neq c}} \right)\neq\sigma\left( \bar{F_{g,c}} \right) \}$ with $\sigma$ the sign function and $\bar{F_{g,c}}$ the log-transformed and set to 0 when unsignificant fold-changes of edgeR for gene g and condition c. This represents genes that are differentially expressed for the considered neoplasm, but not or oppositely for all the other neoplasms. Associated scores were set so their sign follows the regulation direction (positive for up-regulation and negative for down-regulation) and their value follows the amplitude of the maximum difference between up-regulation and down-regulation for the gene, more formally : $S_{c}=\{\sigma\left( \bar{F_{g,c}} \right)\cdot\max_{d\neq c} \left| \bar{F_{g,d}}-\bar{F_{g,c}} \right|:g\in G_{c}$

Each neoplasm specific gene set signature was then subjected to Gene Set Enrichment Analysis (GSEA) to identify pathways that are specifically enriched in each neoplastic component. For this purpose, the WikiPathwayCancer database of the WEB-based GSEA toolkit for Human Transcriptome [www.webgesalt.org](file:///C:\Users\Raphael\AppData\Local\Temp\www.webgesalt.org) was used [14]. Functional enrichment was measured by the normalized enrichment score (NES) and was considered significant when the False Discovery Rates (FDRs) was below 0.05.

A second analysis aimed to identify a reduced set of specific genes that discriminates best neoplasms from each other. This analysis is based on the “gene shaving” method [15] with few adjustments. Partial Least Square-Discriminant Analysis (PLS-DA) was performed on all normalized gene expressions. A small fraction of genes (5%) that contributed the least to the first principal component in terms of absolute values of their coefficients in the PLS-DA regression was iteratively removed or shaved off. This step was repeated until reduction to a set of 5 genes (TOP5) displaying the most specific transcriptomic signature of each neoplasm. This method was used to discriminate each of the three neoplasms from the two others together, and each of the three neoplasms from each other. The script code used is available at <https://github.com/Shiaroku/pls-shaving>.

**Legends of Supplemental Figures**

**Supplemental Figure 1: Pericardial ALK- ALCL.** A. PET-scan imaging revealed pericardial effusion and left ventricle tumoral invasion. B. Pericardial fluid cytological assessment (H&E stained cell block section and Papanicolaou stained ThinPrep smear - inset) showed medium to large atypical lymphocyte with irregular nuclei and dense chromatin associated with numerous apoptotic bodies. By immunohistochemistry, the neoplastic lymphocytes expressed CD2 (C), CD30 (D), granzyme B (E) MUM1/IRF4 (F) and P53 (G). H. TRG PCR identified two dominant peaks, consistent with a clonal rearrangement. Stains original magnification, ×400, H&E - hematoxylin and eosin.

**Supplemental Figure 2. Cytology and phenotype by flow cytometry of acute monoblastic leukemia (AML-M5) in the bone marrow aspirate.** **A.** Wright-Giemsa-stained smear revealed > 90% of large blasts with irregular nuclei, visible nucleoli, and moderate amount of agranulated, pale cytoplasm displaying vacuoles (magnification x1000). **B-E.** By flow cytometry, blastic cells were CD45^+^ (dim) CD33^+^ CD56^+^ CD64^+^ CD117^-^ consistent with acute monoblastic leukemia (AML-M5 by FAB classification).

**Supplemental Figure 3. Spatial transcriptomics of the three hematological malignancies in the lymph node specimen.** Regions of interest (ROI) to quantify RNA expression were manually selected using as guidance scanned hematoxylin and eosin (**a**) and multiplexing immunophenotype using CD68 (yellow), CD20 (green), Ki67 (red) and DNA (blue) (scale bar 2 mm) (**b**). **c** Representative ROIs of non-neoplastic B-lymphoid follicles, AML, DLBCL and ALCL used for transcriptomic analysis. **d** Hierarchical clustering heat map shows distinct clusters of T-, B- cell lymphomas and AML based on their unique gene expression signature. ALCL – anaplastic large cell lymphoma, DLBCL – diffuse large B-cell lymphoma AML – acute myeloid leukemia.

**Supplemental Figure 4. mRNA and protein expression correlation in ALK-ALCL, DLBCL-NOS and AML-M5. A.** Correlation between the protein expression by immunohistochemistry and the mRNA expression in spatial transcriptomics. **B.** Pearson correlation analysis of mRNA and protein expression of p53, IRF4/MUM1, CD20, PAX5, BCL6 and CD56 in ALK- ALCL, DLBCL NOS and AML-M5.

**Supplemental Figure 5. Transcriptomic signatures of the three hematological malignancies A.** Three-dimensional representation of Partial Least Square-Discriminant Analysis (PLS-DA) regression method using only the TOP5 gene list from the gene shaving technique listed in Table 2. Each point represents one sample. **B.** Violin plot representation of mRNA expression of Census genes highlighted by PLS regression method. The three neoplastic components were compared to the non-tumoral group (lymphoid B-follicles and non-neoplastic T-cell paracortex). **: adjusted-p value <0.01 and ***: adjusted-p value <0.001.

**References**

1. Solly, F., F. Angelot-Delettre, M. Ticchioni, F. Genevieve, H. Rambaud, L. Baseggio, A. Plesa, A. Debliquis, F. Garnache-Ottou, A. Roggy, L. Campos, C. Aanei, A. Rosenthal-Allieri, M.T. Georget, S. Lachot, M.C. Jacob, N. Robillard, S. Wuilleme, E. Andre-Kerneis, E. Cornet, V. Salaun, H. Bennami, A.C. Lhoumeau, C. Arnoulet, H. Jacqmin, N. Neyman, V. Latger-Cannard, F. Massin, E. Lainey, M. Le Garff-Tavernier, M. Costopoulos, M. Roussel, C. Mayeur-Rousse, A. Eischen, V. Raggeneau, C. Derrieux, M. Maurer, V. Asnafi, A. Trinquand, C. Brouzes, and L. Lhermitte, *Standardization of Flow Cytometric Immunophenotyping for Hematological Malignancies: The FranceFlow Group Experience.* *Cytometry A*, 2019. 95(9): p. 1008-1018.

2. van Dongen, J.J., A.W. Langerak, M. Bruggemann, P.A. Evans, M. Hummel, F.L. Lavender, E. Delabesse, F. Davi, E. Schuuring, R. Garcia-Sanz, J.H. van Krieken, J. Droese, D. Gonzalez, C. Bastard, H.E. White, M. Spaargaren, M. Gonzalez, A. Parreira, J.L. Smith, G.J. Morgan, M. Kneba, and E.A. Macintyre, *Design and standardization of PCR primers and protocols for detection of clonal immunoglobulin and T-cell receptor gene recombinations in suspect lymphoproliferations: report of the BIOMED-2 Concerted Action BMH4-CT98-3936.* *Leukemia*, 2003. 17(12): p. 2257-317.

3. Li, H. and R. Durbin, *Fast and accurate long-read alignment with Burrows-Wheeler transform.* *Bioinformatics*, 2010. 26(5): p. 589-95.

4. DePristo, M.A., E. Banks, R. Poplin, K.V. Garimella, J.R. Maguire, C. Hartl, A.A. Philippakis, G. del Angel, M.A. Rivas, M. Hanna, A. McKenna, T.J. Fennell, A.M. Kernytsky, A.Y. Sivachenko, K. Cibulskis, S.B. Gabriel, D. Altshuler, and M.J. Daly, *A framework for variation discovery and genotyping using next-generation DNA sequencing data.* *Nat Genet*, 2011. 43(5): p. 491-8.

5. Lai, Z., A. Markovets, M. Ahdesmaki, B. Chapman, O. Hofmann, R. McEwen, J. Johnson, B. Dougherty, J.C. Barrett, and J.R. Dry, *VarDict: a novel and versatile variant caller for next-generation sequencing in cancer research.* *Nucleic Acids Res*, 2016. 44(11): p. e108.

6. McLaren, W., L. Gil, S.E. Hunt, H.S. Riat, G.R. Ritchie, A. Thormann, P. Flicek, and F. Cunningham, *The Ensembl Variant Effect Predictor.* *Genome Biol*, 2016. 17(1): p. 122.

7. Greenberg, P.L., R.M. Stone, A. Al-Kali, J.M. Bennett, U. Borate, A.M. Brunner, W. Chai-Ho, P. Curtin, C.M. de Castro, H.J. Deeg, A.E. DeZern, S. Dinner, C. Foucar, K. Gaensler, G. Garcia-Manero, E.A. Griffiths, D. Head, B.A. Jonas, S. Keel, Y. Madanat, L.J. Maness, J. Mangan, S. McCurdy, C. McMahon, B. Patel, V.V. Reddy, D.A. Sallman, R. Shallis, P.J. Shami, S. Thota, A.N. Varshavsky-Yanovsky, P. Westervelt, E. Hollinger, D.A. Shead, and C. Hochstetler, *NCCN Guidelines(R) Insights: Myelodysplastic Syndromes, Version 3.2022.* *J Natl Compr Canc Netw*, 2022. 20(2): p. 106-117.

8. Dohner, H., A.H. Wei, F.R. Appelbaum, C. Craddock, C.D. DiNardo, H. Dombret, B.L. Ebert, P. Fenaux, L.A. Godley, R.P. Hasserjian, R.A. Larson, R.L. Levine, Y. Miyazaki, D. Niederwieser, G. Ossenkoppele, C. Rollig, J. Sierra, E.M. Stein, M.S. Tallman, H.F. Tien, J. Wang, A. Wierzbowska, and B. Lowenberg, *Diagnosis and management of AML in adults: 2022 recommendations from an international expert panel on behalf of the ELN.* *Blood*, 2022. 140(12): p. 1345-1377.

9. Sujobert, P., Y. Le Bris, L. de Leval, A. Gros, J.P. Merlio, C. Pastoret, S. Huet, C. Sarkozy, F. Davi, M. Callanan, C. Thieblemont, D. Sibon, V. Asnafi, C. Preudhomme, P. Gaulard, F. Jardin, G. Salles, and E. Macintyre, *The Need for a Consensus Next-generation Sequencing Panel for Mature Lymphoid Malignancies.* *Hemasphere*, 2019. 3(1): p. e169.

10. Okutman, O., J. Tarabeux, J. Muller, and S. Viville, *Evaluation of a Custom Design Gene Panel as a Diagnostic Tool for Human Non-Syndromic Infertility.* *Genes (Basel)*, 2021. 12(3).

11. Merritt, C.R., G.T. Ong, S.E. Church, K. Barker, P. Danaher, G. Geiss, M. Hoang, J. Jung, Y. Liang, J. McKay-Fleisch, K. Nguyen, Z. Norgaard, K. Sorg, I. Sprague, C. Warren, S. Warren, P.J. Webster, Z. Zhou, D.R. Zollinger, D.L. Dunaway, G.B. Mills, and J.M. Beechem, *Multiplex digital spatial profiling of proteins and RNA in fixed tissue.* *Nat Biotechnol*, 2020. 38(5): p. 586-599.

12. Dottermusch, M., Y. Schumann, U. Kordes, M. Hasselblatt, and J.E. Neumann, *Spatial molecular profiling of a central nervous system low-grade diffusely infiltrative tumour with INI1 deficiency featuring a high-grade atypical teratoid/rhabdoid tumour component.* *Neuropathol Appl Neurobiol*, 2022. 48(3): p. e12777.

13. Costa-Silva, J., D. Domingues, and F.M. Lopes, *RNA-Seq differential expression analysis: An extended review and a software tool.* *PLoS One*, 2017. 12(12): p. e0190152.

14. Liao, Y., J. Wang, E.J. Jaehnig, Z. Shi, and B. Zhang, *WebGestalt 2019: gene set analysis toolkit with revamped UIs and APIs.* *Nucleic Acids Res*, 2019. 47(W1): p. W199-W205.

15. Hastie, T., R. Tibshirani, M.B. Eisen, A. Alizadeh, R. Levy, L. Staudt, W.C. Chan, D. Botstein, and P. Brown, *'Gene shaving' as a method for identifying distinct sets of genes with similar expression patterns.* *Genome Biol*, 2000. 1(2): p. RESEARCH0003.
