# Supplementary figures and images for: Multiomics of three hematological malignancies in a patient reveal their origin from clonal hematopoietic stem cells
Source: Blood Cancer J. 2023 Aug 9;13(1):118. doi: 10.1038/s41408-023-00892-w (PMC10412639; doi:10.1038/s41408-023-00892-w)

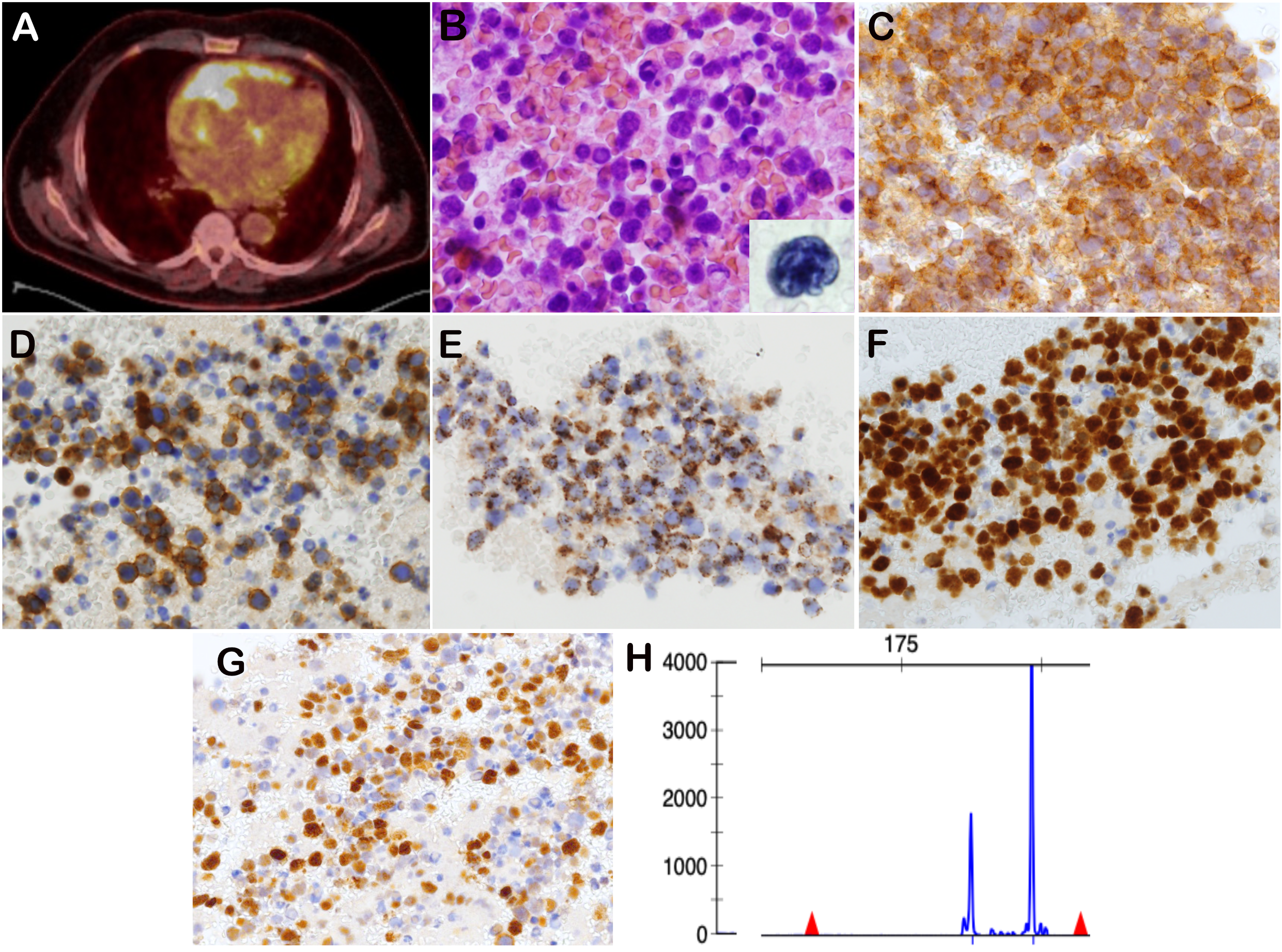

Supplement: Supplementary file 2 — Supplemental Figure 1 [file 41408_2023_892_MOESM2_ESM.tif]

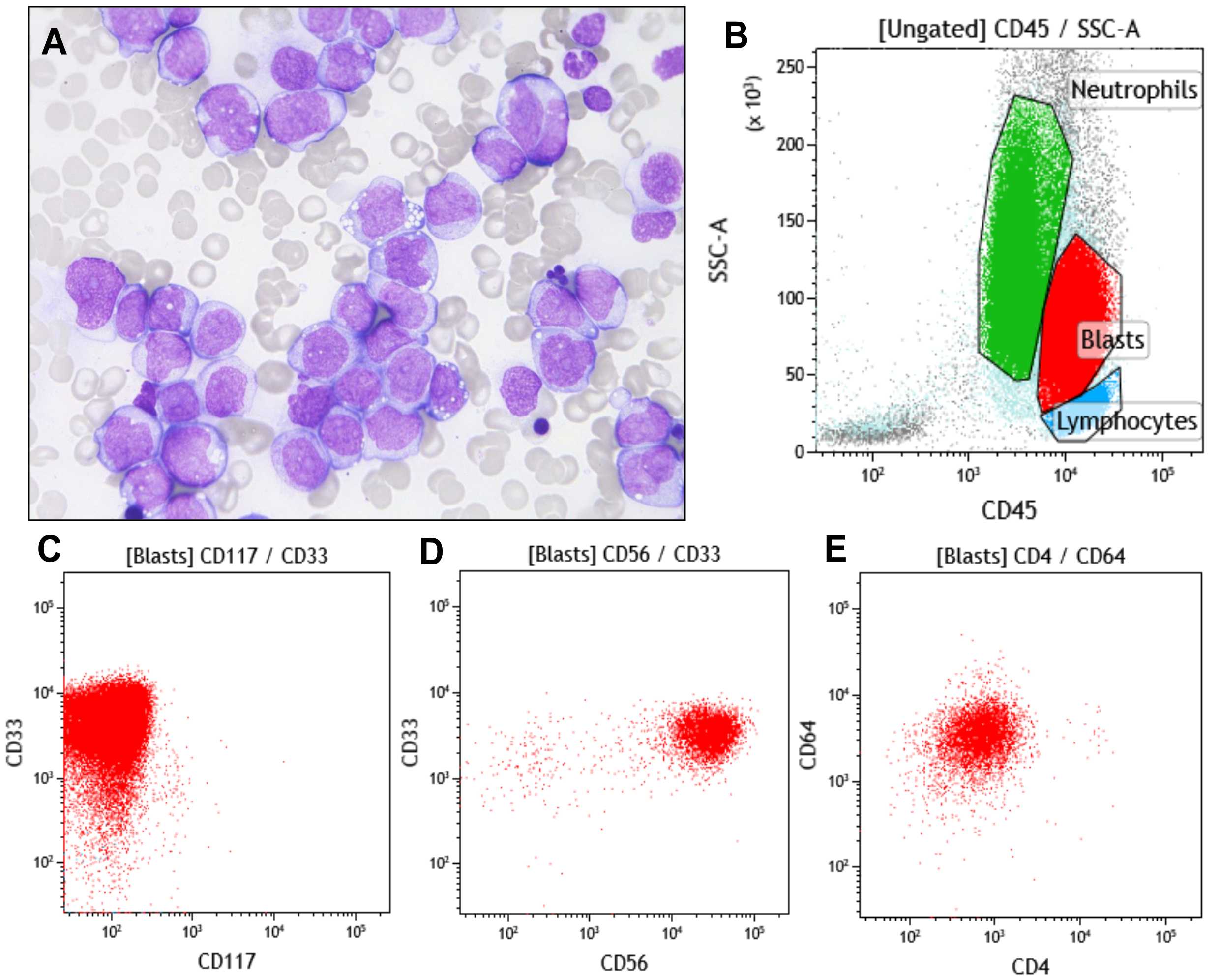

Supplement: Supplementary file 3 — Supplemental Figure 2 [file 41408_2023_892_MOESM3_ESM.tif]

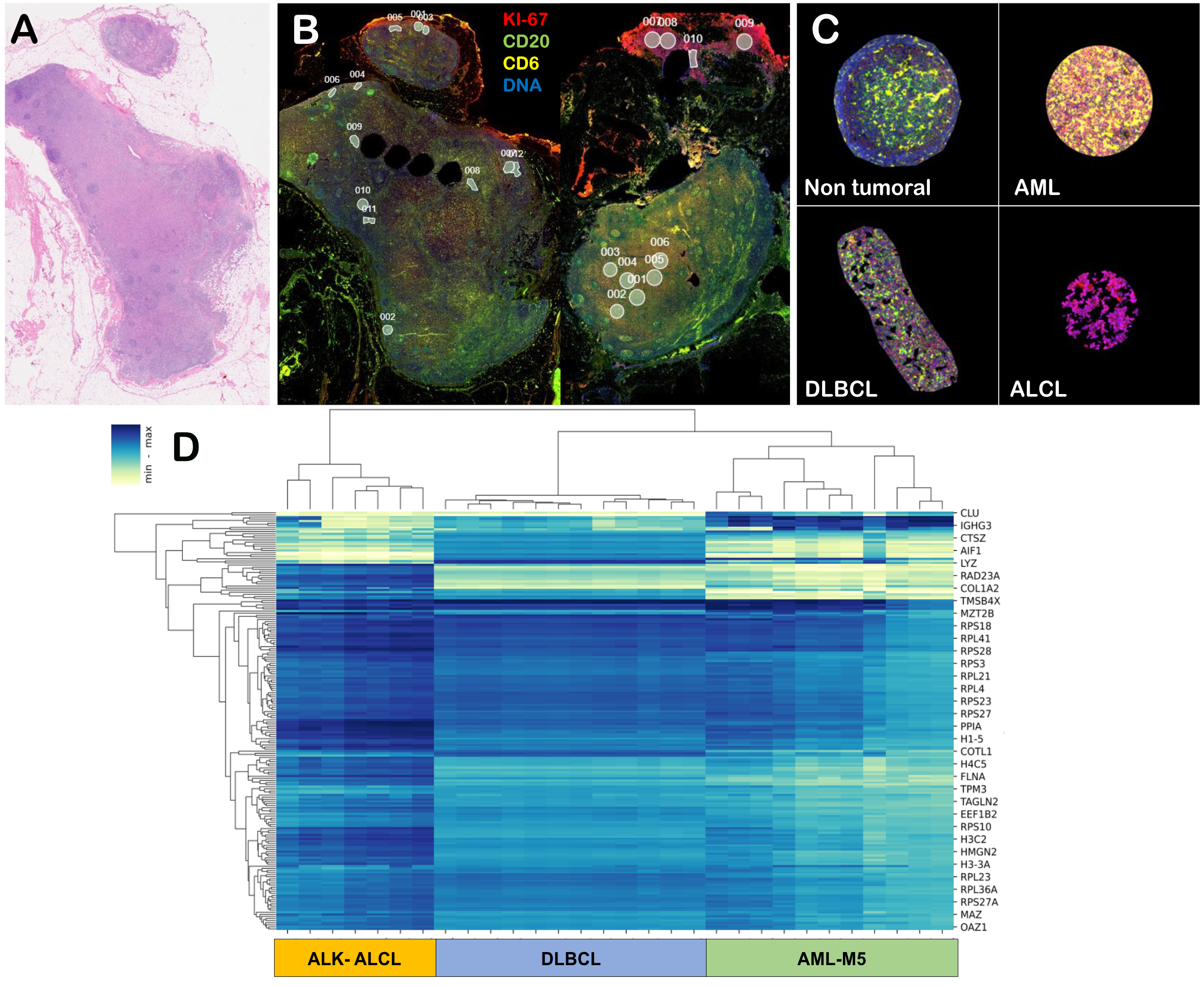

Supplement: Supplementary file 4 — Supplemental Figure 3 [file 41408_2023_892_MOESM4_ESM.tif]

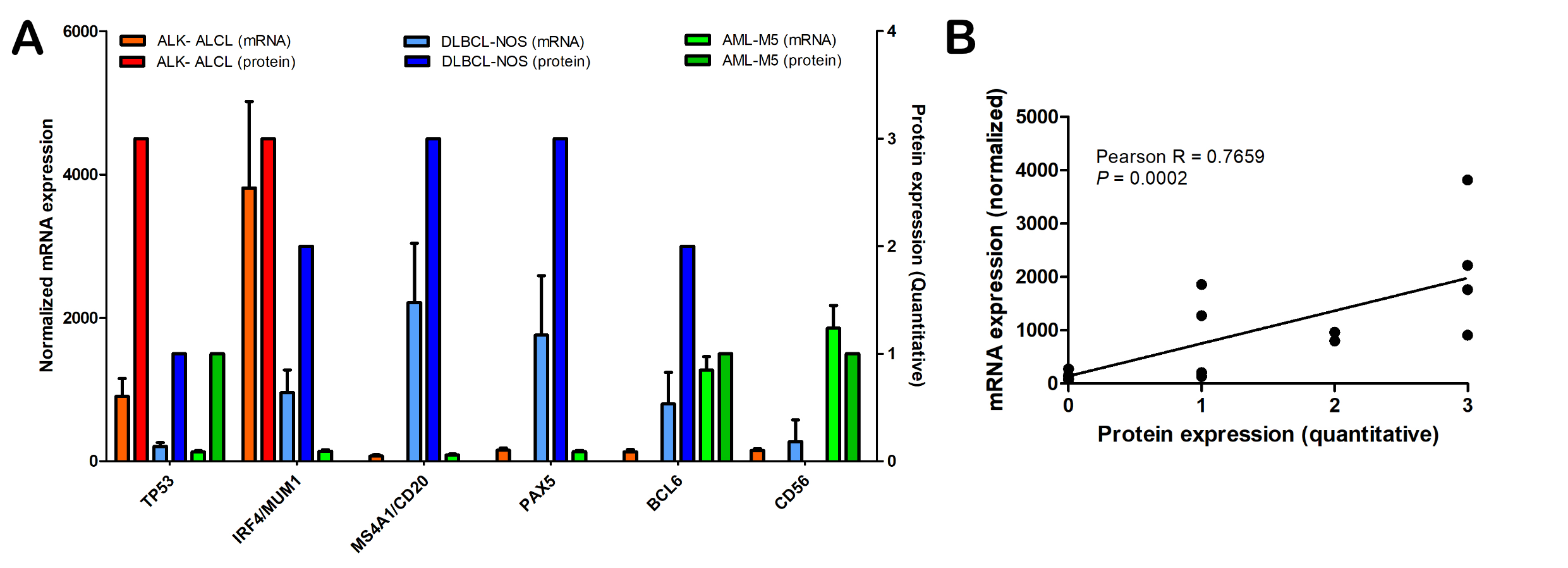

Supplement: Supplementary file 5 — Supplemental Figure 4 [file 41408_2023_892_MOESM5_ESM.tif]

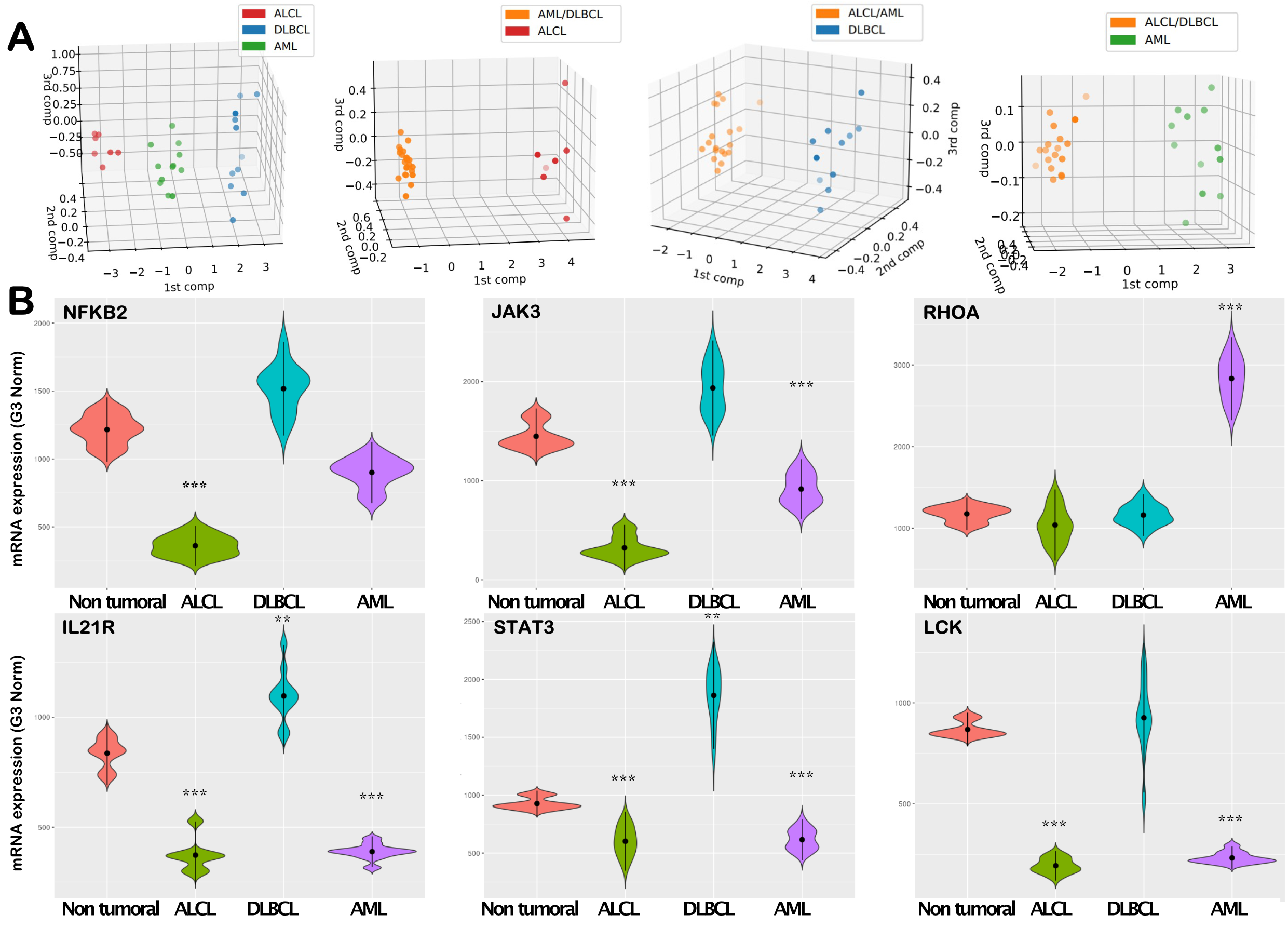

Supplement: Supplementary file 6 — Supplemental Figure 5 [file 41408_2023_892_MOESM6_ESM.tif]
